# Single-System Laryngeal Anaphylaxis Following Gadobutrol Administration

**DOI:** 10.7759/cureus.113693

**Published:** 2026-07-30

**Authors:** Menooa Simonian, Masoud Nakhaei, Bruno Panzarini

**Affiliations:** 1 Chicago Medical School, Rosalind Franklin University of Medicine and Science, North Chicago, USA; 2 Neuroradiology, University of California San Diego, La Jolla, USA

**Keywords:** anaphylaxis, cough, gadolinium-based contrast agents, hoarseness, laryngeal edema, mri adverse reactions

## Abstract

Gadolinium-based contrast agents (GBCAs) are widely used in magnetic resonance imaging (MRI) and are generally considered safe, with immediate hypersensitivity reactions being rare. While anaphylaxis typically presents with classic skin reactions, respiratory distress, or cardiovascular collapse, exposure to a known allergen can cause single-system respiratory or laryngeal variants. An 83-year-old male with multiple chronic medical conditions underwent a diagnostic brain MRI with contrast. Immediately after receiving 8.5 mL of gadobutrol, the patient developed hoarseness and a sporadic cough. Notably, he had no skin reactions, dyspnea, tachypnea, drop in oxygen saturation, or hypotension. Instead, his blood pressure remained stable but elevated. Due to the rapid onset of these isolated upper airway symptoms, a rapid response was triggered. He was treated proactively with epinephrine, diphenhydramine, and methylprednisolone, and transferred to the intensive care unit (ICU) for close monitoring. Bedside flexible laryngoscopy confirmed only minimal right arytenoid edema and a patent glottis. He completely recovered within 24 hours without further clinical progression. This case underscores a highly atypical presentation of GBCA-induced anaphylaxis localized entirely to the upper airway, manifesting solely as hoarseness and coughing. As an isolated respiratory reaction to a known non-inhaled allergen, this case shows how the absence of classic multisystem involvement can easily mask a severe allergic reaction.

## Introduction

Gadolinium-based contrast agents (GBCAs) are essential to modern diagnostic imaging and maintain an excellent safety profile. Immediate hypersensitivity reactions are exceedingly rare and typically non-IgE-mediated (anaphylactoid). A landmark systematic review and meta-analysis found that the overall pooled rate of immediate allergic-like contrast reactions to GBCAs is approximately 9.2 per 10,000 administrations (0.092%), with severe, life-threatening events occurring at a rate of 0.52 per 10,000 administrations (0.0052%) [1].

Contrast reactions are traditionally classified by the American College of Radiology (ACR) Manual on Contrast Media as mild, moderate, or severe based on clinical severity. Under the ACR framework, hoarseness without dyspnea or other signs of distress and hemodynamic collapse fall into the category of a Moderate Allergic-Like reaction [2]. Distinguishing this from single-system anaphylaxis (such as under GA²LEN criteria) is critical in this context, as isolated upper airway involvement carries an inherent risk of rapid, unpredictable occlusion that warrants immediate epinephrine despite initial hemodynamic stability.

As outlined in the Anaphylaxis Clinical Support Tool from the 2024 GA²LEN consensus report, anaphylaxis is considered likely when any one of three distinct criteria is met: (1) with “No Known Allergen Exposure,” a sudden onset of an illness (minutes to several hours) with skin/mucosal involvement and either respiratory or cardiovascular involvement; (2) with a “Likely or Known Allergen Exposure,” a sudden onset of two or more of skin/mucosal involvement, respiratory involvement, cardiovascular involvement, or severe gastrointestinal involvement; or (3) with a “Known Allergen Exposure,” a sudden onset of either respiratory involvement after exposure to a non-inhaled allergen, or cardiovascular involvement [3].

Recognizing single-system variants under this third pathway is challenging, particularly when a patient presents with no obvious respiratory distress, clear lungs, and elevated blood pressure despite localized upper airway symptoms. This case report highlights an 83-year-old male who experienced gadolinium-induced anaphylaxis presenting only with hoarseness and coughing. This case illustrates how isolated respiratory symptoms following a known, non-inhaled allergen exposure can fulfill modern anaphylaxis criteria in the absence of overt systemic distress.

## Case presentation

An 83-year-old male was admitted to the hospital for evaluation of a three-day history of intermittent, positional dizziness. His medical history included hypertension, hyperlipidemia, peripheral vascular disease, benign prostatic hyperplasia, chronic bladder symptoms, gastroesophageal reflux disease, migraine headaches, restless legs syndrome, and obstructive sleep apnea. None of his home medications are known to mask signs of anaphylaxis. The patient previously tolerated iodinated contrast media without issue, and his known baseline drug allergies included procaine (causing dizziness and shortness of breath), simvastatin (causing weakness and muscle pain), lovastatin (causing muscle pain), lidocaine (allergic response, type unknown), tamsulosin (causing dizziness), and meloxicam (causing insomnia). He drank alcohol occasionally for religious services and had no history of tobacco use.

His initial workup for dizziness, including a non-contrast head CT, head and neck CT angiography (CTA), orthostatic vitals, chest X-ray, and an acute coronary syndrome (ACS) evaluation, showed no acute issues.

On day 2, new-onset atrial fibrillation with rapid ventricular response (RVR) triggered emergent evaluation by the rapid response team. It was controlled with a single 5 mg intravenous (IV) dose of metoprolol followed by metoprolol tartrate 25 mg p.o. every 12 hours PRN. The patient felt completely fine throughout the event.

On hospital day 3, a brain MRI with and without contrast was performed. The patient received 8.5 mL of IV gadobutrol (Gadavist; Bayer HealthCare, Whippany, USA). Within minutes, he began coughing, and MR personnel noticed a change in the patient’s voice, which prompted emergency evaluation by an on-site physician. 

Crucially, the patient experienced no respiratory distress. He denied any shortness of breath, chest tightness, throat narrowing, or difficulty swallowing. His vitals revealed an elevated blood pressure of 168/87 mmHg (peaking at 180/90 mmHg), a stable heart rate of 58 beats per minute in sinus bradycardia, an unlabored respiratory rate of 20 breaths per minute, and an oxygen saturation of 100% on room air. Skin examinations demonstrated a complete absence of hives, maculopapular rashes, erythema, pruritus, or cyanosis. There was no visible swelling of the tongue, lips, or uvula, and no pooling of oral secretions. Pulmonary auscultation revealed no loud stridor, no intercostal retractions, and no use of accessory muscles, confirming that air movement was completely preserved. 

The clinical team recognized that sudden-onset hoarseness and coughing post-GBCA injection could represent early, localized laryngeal involvement. To prevent rapid airway compromise, a rapid response was triggered. The patient was managed with a 0.3 mg intramuscular epinephrine injection, followed by a secondary dose of 0.3 mg intravenously during transport. The secondary dose (0.3 mg IV) was administered during transport to the ICU as a proactive measure to prevent rapid, unpredictable airway closure while en route, prior to definitive bedside flexible laryngoscopy and airway visualization. Two doses of intravenous diphenhydramine 25 mg, an intravenous push of 125 mg methylprednisolone, and one liter of normal saline were also administered. He was upgraded to the intensive care unit (ICU) for further monitoring.

Otolaryngology was consulted urgently in the ICU to perform a definitive structural evaluation of the upper airway. A bedside flexible laryngoscopy revealed a completely patent glottis, minimal right arytenoid edema, and no significant vocal cord swelling or mechanical airway obstruction, confirming there were no indications for endotracheal intubation. 

The patient's hoarseness and cough completely resolved over the subsequent 24 hours. To manage his acute post-allergic course, he was started on prednisone 40 mg p.o. daily, cetirizine 10 mg p.o. daily, and albuterol 3 mg/ipratropium 0.5 mg per 3 mL solution on a temporary as-needed basis.

The comprehensive findings of all the laboratory investigations on the day of the acute reaction are systematically compiled in Table 1.

**Table 1:** Laboratory Values pCO2: partial pressure of carbon dioxide; HCO3: bicarbonate

Parameters	Patient Values	Reference range
Troponin	19.0 ng/L	< 14.0 ng/L
Acute Serum Tryptase (2 hours post-reaction)	8.1 mcg/L	< 11.4 mcg/L
Baseline Serum Tryptase (25 hours post-reaction)	5.8 mcg/L	< 11.4 mcg/L
White Blood Cell (WBC) Count	7.26 x 10^3/uL	4.5 - 11.0 x 10^3/uL
Hemoglobin	14.0 g/dL	13.8 - 17.2 g/dL
Platelets	158 x 10^3/uL	150 - 450 x 10^3/uL
Potassium	3.9 mEq/L	3.5 - 5.0 mEq/L
Blood Urea Nitrogen (BUN)	26 mg/dL	8 - 20 mg/dL
Creatinine	0.87 mg/dL	0.70 - 1.30 mg/dL
Venous Blood Gas (VBG) pH	7.31	7.31 - 7.41
VBG pCO2	57 mmHg	40 - 52 mmHg
VBG HCO3	29 mEq/L	22 - 29 mEq/L

Electrocardiogram (EKG) showed sinus rhythm with no acute ST-segment changes. Imaging included a grossly stable chest X-ray noting possible interval left basilar opacities, and an MRI of the brain with contrast, which revealed no acute intracranial abnormalities.

## Discussion

Immediate hypersensitivity reactions to GBCAs like gadobutrol are exceptionally rare. Current global standards defined by the 2024 GA²LEN Anaphylaxis Clinical Support Tool formally establish that a known allergen exposure resulting in the sudden onset of isolated respiratory involvement after exposure to a non-inhaled allergen satisfies the definitive diagnostic criteria for anaphylaxis. As detailed in the Anaphylaxis Clinical Support Tool, respiratory involvement explicitly encompasses laryngeal signs such as voice changes, alongside symptoms like cough [3]. 

Despite matching these diagnostic criteria, the presentation was intriguing due to the complete absence of systemic distress. In classic emergency medicine teaching, respiratory involvement during anaphylaxis is synonymous with clear distress signals such as tachypnea, accessory muscle use, retractions, subjective air hunger, stridor, or drops in oxygen saturation. Our patient exhibited none of these signs.

A critical lesson from this case lies in the distinction between radiology-specific classification systems and global allergy criteria. Under the ACR Manual on Contrast Media, acute contrast reactions manifesting as hoarseness without dyspnea or other signs of distress and hemodynamic collapse are classified as Moderate Allergic-Like reactions [2]. Conversely, under the GA²LEN consensus framework detailed above, isolated upper airway symptoms following exposure to a known parenteral agent satisfy the definition of single-system anaphylaxis [3].

Regardless of whether the event is categorized as an ACR Moderate Allergic-Like reaction or single-system anaphylaxis, the clinical decision to administer immediate intramuscular epinephrine was vital. Laryngeal edema induced by intravenous contrast agents can progress insidiously. Delaying treatment until overt stridor, severe dyspnea, or cardiovascular collapse occurs - which would formally shift the ACR classification to “severe”' - puts the patient at risk for rapid airway obstruction.

Early epinephrine administration remains the cornerstone of upper airway stabilization, reversing early laryngeal edema before complete airway occlusion occurs. The clinical team's immediate intervention prioritized airway safety over delayed observation, successfully halting symptom progression.

The patient's objective biomarker data provide additional context for the immunologic nature of this event, though interpretation requires careful consideration of several limitations. The initial serum tryptase level of 8.1 mcg/L did not exceed the commonly cited laboratory threshold of 11.4 mcg/L for systemic anaphylaxis. However, expert consensus from the 2023 anaphylaxis practice parameter update recommends that an acute serum tryptase level at least 20% + 2 ng/mL above the patient's baseline serum tryptase is more sensitive evidence of systemic mast cell activation than a fixed laboratory cutoff alone. Using the follow-up serum tryptase of 5.8 mcg/L drawn the next day as a baseline to calculate, the threshold would be (1.2 x 5.8 mcg/L) + 2 mcg/L = 8.96 mcg/L. The post-reaction value of 8.1 mcg/L still falls below this threshold, and the acute-to-baseline ratio of 1.4 does not reach the alternative ratio threshold of 1.685 proposed by the criteria. However, several factors suggest this calculation likely underestimates the true magnitude of mast cell activation. First, the initial sample was drawn approximately two hours after the symptom's onset. Because serum tryptase concentration peaks within 30 to 60 minutes of the reaction and then declines with an approximate half-life of two hours, the initial draw likely captured a declining phase rather than the true physiological peak. Second, the patient had already received epinephrine, diphenhydramine, and methylprednisolone prior to the blood draw, all of which may have attenuated the ongoing mast cell response. Third, the decline in trajectory from 8.1 to 5.8 mcg/L is consistent with the kinetic pattern of acute mast cell degranulation, even if the absolute value doesn't meet the formal thresholds. Importantly, the 2023 practice parameter update emphasizes that a lack of tryptase elevation should not be interpreted as ruling out a diagnosis of anaphylaxis. Tryptase levels are frequently normal in non-parenteral and milder anaphylactoid reactions, and the clinical diagnosis of anaphylaxis remains fundamentally based on the presenting signs and symptoms rather than on biomarker confirmation alone [4]. 

While serum tryptase was drawn in the acute setting, formal outpatient allergy consultation and contrast skin testing were not scheduled, as management prioritized patient counseling on strict future GBCA class avoidance and pre-procedure prophylaxis protocols.

Furthermore, the patient presented with two additional major confounding factors. The first was the absence of cutaneous manifestations. This patient had completely clear skin, eliminating the most common visual trigger for an allergic workup. The second factor was persistent, paradoxical hypertension. Instead of standard anaphylactic shock marked by profound distributive hypotension, this patient maintained systolic blood pressures up to 180 mmHg. While hypotension is a classic diagnostic anchor, a distinct "hypertensive anaphylaxis" phenotype is increasingly recognized in acute care settings, with a reported prevalence of up to 12.9% of anaphylaxis presentations [5]. This paradoxical hypertension is driven by intense compensatory sympathetic activation, endogenous catecholamine surge, or anxiety [6]. Over-reliance on a blood pressure drop to diagnose anaphylaxis in these cases frequently delays life-saving care.

The pathophysiological mechanism of immediate reactions to GBCAs is largely non-IgE mediated, resulting from direct activation of the complement system or direct mast cell degranulation rather than prior immunologic sensitization [7]. This explains why the patient had successfully tolerated past contrast-enhanced MRIs without incident. The immediate onset of localized right arytenoid edema - confirmed via flexible laryngoscopy - proves that the hoarseness was a structural warning sign of early upper airway edema rather than an isolated benign event. Because the patient wasn’t in respiratory distress, it would have been easy to misattribute his symptoms to a mild transient laryngeal spasm or an acute exacerbation of gastroesophageal reflux disease. The clinical team's decision to treat these isolated symptoms aggressively was critical to avoid progression into catastrophic, occlusive laryngeal edema.

The objective demonstration of acute, localized arytenoid edema on flexible laryngoscopy - combined with the immediate temporal relationship to gadobutrol administration and rapid resolution following epinephrine - clinically excludes functional airway disorders (such as paradoxical vocal fold motion, transient vocal cord dysfunction, or anxiety-induced dysphonia) as well as non-allergic adduction mechanisms (laryngospasm). Furthermore, alternative diagnoses such as ACE-inhibitor angioedema or recurrent laryngeal nerve pathology are clearly incongruent with the clinical timeline and localized physical findings.

## Conclusions

This case demonstrates that gadobutrol-induced hypersensitivity can present insidiously, isolated entirely to vocal changes and coughing without overt respiratory or cardiovascular collapse, or cutaneous signs. While classified as a Moderate Allergic-Like reaction under ACR criteria, this presentation satisfies modern GA²LEN diagnostic criteria for single-system anaphylaxis. In the setting of post-contrast monitoring, localized hoarseness can represent early upper airway edema; recognizing this early structural sign rather than awaiting classic multisystem deterioration remains critical to preventing sudden airway compromise.
